# Silver Nanoparticles Induced Changes in DNA Methylation and Histone H3 Methylation in a Mouse Model of Breast Cancer

**DOI:** 10.3390/ma16114163

**Published:** 2023-06-02

**Authors:** Kamil Brzóska, Barbara Sochanowicz, Małgorzata Szczygieł, Agnieszka Drzał, Martyna Śniegocka, Dominika Michalczyk-Wetula, Martyna Elas, Lucyna Kapka-Skrzypczak, Marcin Kruszewski

**Affiliations:** 1 Centre for Radiobiology and Biological Dosimetry, Institute of Nuclear Chemistry and Technology, Dorodna 16, 03-195 Warsaw, Poland; 2 Department of Biophysics and Cancer Biology, Faculty of Biochemistry, Biophysics and Biotechnology, Jagiellonian University, Gronostajowa 7, 30-387 Cracow, Poland; 3 Department of Molecular Biology and Translational Research, Institute of Rural Health, Jaczewskiego 2, 20-090 Lublin, Poland; 4 World Institute for Family Health, Calisia University, 62-800 Kalisz, Poland

**Keywords:** silver nanoparticles, AgNPs, DNA methylation, histone modifications, epigenetics

## Abstract

The importance of epigenetic changes as a measurable endpoint in nanotoxicological studies is getting more and more appreciated. In the present work, we analyzed the epigenetic effects induced by citrate- and PEG-coated 20 nm silver nanoparticles (AgNPs) in a model consisting of 4T1 breast cancer tumors in mice. Animals were administered with AgNPs intragastrically (1 mg/kg b.w. daily—total dose 14 mg/kg b.w.) or intravenously (administration twice with 1 mg/kg b.w.—total dose 2 mg/kg b.w.). We observed a significant decrease in 5-methylcytosine (5-mC) level in tumors from mice treated with citrate-coated AgNPs regardless of the route of administration. For PEG-coated AgNPs, a significant decrease in DNA methylation was observed only after intravenous administration. Moreover, treatment of 4T1 tumor-bearing mice with AgNPs decreased histone H3 methylation in tumor tissue. This effect was the most pronounced for PEG-coated AgNPs administered intravenously. No changes in histone H3 Lys9 acetylation were observed. The decrease in methylation of DNA and histone H3 was accompanied by changes in expression of genes encoding chromatin-modifying enzymes (Setd4, Setdb1, Smyd3, Suv39h1, Suv420h1, Whsc1, Kdm1a, Kdm5b, Esco2, Hat1, Myst3, Hdac5, Dnmt1, Ube2b, and Usp22) and genes related to carcinogenesis (Akt1, Brca1, Brca2, Mlh1, Myb, Ccnd1, and Src). The significance of the observed changes and the mechanisms responsible for their development are unclear, and more research in this area is warranted. Nevertheless, the present work points to the epigenetic effects as an important level of interaction between nanomaterials and biological systems, which should always be taken into consideration during analysis of the biological activity of nanomaterials and development of nanopharmaceuticals.

## 1. Introduction

For the past 15 years, there has been a significant amount of research focused on the interaction mechanisms between biological systems and silver nanoparticles (AgNPs), as well as their potential toxicity. The reason for this continuing interest of scientists in this research topic is the widespread use of AgNPs in industry and medicine rising justified safety concerns. Initial research was focused on such endpoints as cell viability, survival and proliferation, DNA damage, oxidative stress, apoptosis induction, and cell cycle disturbance [1]. Progressively, other aspects of the cellular machinery involved in response to AgNPs were analyzed, such as gene expression and activity of intracellular signaling pathways [2,3]. Biological models used in silver nanotoxicology studies also shifted from simple 2D cell cultures and normal, healthy animals, to more sophisticated 3D in vitro models and animal models allowing analysis of AgNPs’ impact on developing embryos or on the progression of the tumorigenesis process [4,5,6,7,8,9]. The latter aspect is especially important in the context of the potential anti-cancer activity of AgNPs, which was reported by many authors, but mostly based on data from in vitro experiments [10,11,12,13]. All of the nanotoxicological studies performed so far resulted in a general understanding of the mechanism of action of AgNPs in biological systems, pointing to the induction of oxidative stress inside cells as a fundamental mechanism responsible for nanoparticle toxicity [14,15]. Over time, it has become increasingly evident that nanoparticle exposure contributes to biological effects through epigenetic alterations, and the importance of epigenetic changes as a measurable endpoint in nanotoxicology studies is getting more and more appreciated [16,17]. The chromatin structure is determined by epigenetic mechanisms, which encompass DNA methylation, histone modifications, and non-coding RNAs. For example, histone modifications have a profound impact on the structure of chromatin associated with the formation of transcriptionally active and inactive regions of the genome. Methylation of histone 3 has been demonstrated to activate the gene expression when it occurs at lysine 4, 36, and 79 or restrain gene expression at lysine 9 and 27 [18]. Studies have demonstrated that AgNPs can alter DNA methylation and histone H3 modification status and influence the expression of non-coding RNA. It was postulated that epigenetic changes may serve as a very sensitive and reliable marker of exposure to nanomaterials, including AgNPs [16,17,19,20,21].

Our recent study involved an investigation of how AgNPs, in terms of their coating and administration route, affect the development and metastatic potential of 4T1 breast cancer in BALB/ccmdb immunocompetent mice. It was found that even though AgNPs administration did not affect tumor growth kinetics, it significantly inhibited metastasis formation [22]. The effect was evidently reliant on both the coating of nanoparticles and the administration route since it was observed only after intragastric administration of citrate-coated but not PEG-coated AgNPs. Despite the suppression of metastatic potential, there were no alterations detected in the expression of genes linked to epithelial-mesenchymal transition (EMT). Therefore, we hypothesized that the effect was not due to the direct action of AgNPs on cancer cells, but rather a consequence of immune system regulation. The present work expands the previous study with an analysis of epigenetic changes in 4T1 tumors from mice treated with AgNPs. It shows that AgNPs treatment affected the tumor’s global DNA methylation and histone H3 methylation. This was accompanied by changes in the expression of chromatin modification enzymes, as well as oncogenes and tumor suppressor genes.

## 2. Materials and Methods

### 2.1. Animals and Treatment

A detailed description of the animal model used in the study is given in the previous paper [22]. Briefly, 3-month-old female BALB/ccmdb mice were subcutaneously injected into the mammary fat pad with 1 × 10^5^ 4T1 cells suspended in 100 µL of PBS. The tumor became apparent 5 days following inoculation. Citrate-coated AgNPs (20 nm nominal size) were purchased from NanoComposix (San Diego, CA, USA). According to the manufacturer’s data, the diameter measured by TEM was 19.9 ± 2.8 nm while hydrodynamic diameter was 25 nm. Zeta potential was equal to −43 mV. PEG-coated AgNPs were obtained from citrate-coated AgNPs as described previously [22]. AgNPs were diluted in sterile water and administered to animals at a dose of 1 mg/kg. To simulate two main roads of administration of possible nanoparticle-based anti-cancer agents, AgNPs were administered intragastrically or intravenously through tail vein injection. Control animals received water. In the original study [22], six animals per experimental group were used. However, due to technical problems (the amount of material from some animals was insufficient to perform all analyses planned in the project) and budget limitations, the epigenetic analyses described in the present work were performed on samples from three animals per group.

### 2.2. ELISA

Histone extraction from tumor tissue samples was carried out using EpiQuik ™ Total Extraction Kit (Epigentec, Farmingdale, NY, USA). Total protein concentration was determined with the Bradford method [23]. Histone H3 acetylated Lys9, Histone H3 methylated Lys4, Lys9, and Lys27 were assayed using dedicated ELISA kits (Active Motif Inc., Carlsbad, CA, USA) following manufacturer’s protocols. 

DNA was extracted from tumor tissue samples using DNeasy Blood & Tissue Kit (Qiagen, Hilden, Germany) using a protocol provided by the manufacturer. Global DNA methylation occurring as the formation of 5-methylcytosine (5-mC) was determined using MethylFlash Global DNA Methylation (5-mC) ELISA Easy Kit (Colorimetric) (Epigentec, Farmingdale, NY, USA) according to manufacturer’s guidelines.

### 2.3. Analysis of miRNA and mRNA Expression by Real-Time PCR

Total RNA including miRNA was extracted from tumor tissue samples using a miRNeasy Mini Kit (Qiagen, Hilden, Germany). The QuantiFluor RNA System (Promega, Madison, WI, USA) and Quantus Fluorometer (Promega, Madison, WI, USA) were used to determine RNA concentration.

For miRNA expression analysis, 250 ng of RNA was reverse transcribed using miScript II RT Kit (Qiagen, Hilden, Germany). cDNA samples were diluted to 200 µL with nuclease-free water and submitted to real-time PCR analysis, which was performed in a 25 µL reaction mixture containing 2.5 µL of diluted cDNA, 12.5 µL QuantiTect SYBR Green PCR Master Mix (Qiagen, Hilden, Germany), 2.5 µL of mi Script Universal Primer (Qiagen, Hilden, Germany), 2.5 µL miScript Primer Assay (Qiagen, Hilden, Germany), and 5 µL H_2_O. The following miScript Primer Assays were used: Mm_let-7f_1 (Mirlet7f-1), Mm_miR-29a*_2 (Mir29a), Mm_miR-199a-5p_1 (Mir199a-1), Mm_miR-103_2 (Mir103-1), Mm_let-7a_2 (Mirlet7a-1), Mm_miR-9_1 (Mir9-1), Mm_miR-21_2 (Mir21), Mm_miR-200b_3 (Mir200b), Mm_miR-17_1 (Mir17), Mm_let-7g_2 (Mirlet7g), Mm_miR-200c_1 (Mir200c), Mm_miR-200a_1 (Mir200a), Mm_miR-93_1 (Mir93), Hs_SNORD95_11 (SNORD95). PCR amplification was performed using a 7500 Real-Time PCR System (Applied Biosystems, Thermo Fisher Scientific, Waltham, MA, USA). PCR conditions were as follows: initial 15 min step at 95 °C, 40 cycles of 94 °C for 15 s, 55 °C for 30 s, and 70 °C for 34 s. The ΔΔCt method was employed, with SNORD95 as endogenous control, to determine the relative expression of miRNA.

For mRNA expression analysis, 1 µg of total RNA was reverse-transcribed to cDNA using iScript Advanced cDNA Synthesis Kit (Bio-Rad, Hercules, CA, USA). cDNA was diluted to 105 µL with nuclease-free water and subjected to real-time PCR analysis using Epigenetic Chromatin Modification Enzymes PCR Array, and Oncogenes and tumor suppressor genes PCR Array (Bio-Rad, Hercules, CA, USA). PCR amplification was performed using a 7500 Real-Time PCR System (Applied Biosystems, Thermo Fisher Scientific, Waltham, MA, USA). The following PCR conditions were employed: initial 2 min step at 95 °C, 40 cycles of 95 °C for 5 s, and 60 °C for 30 s. The ΔΔCt method was employed, with Actb, Gusb, and Hprt as endogenous controls, to determine the relative mRNA expression [24].

Relative Quantification Software version 2021.1.1-Q1-21-build11 (Thermo Fisher Cloud, Thermo Fisher Scientific, Waltham, MA, USA) was used for all calculations.

### 2.4. Statistical Evaluation

Statistica 7.1 software (StatSoft, Tulsa, OK, USA) was used for statistical analysis. The statistical significance of differences was evaluated using Student’s *t*-test and Mann–Whitney U test, where a *p* < 0.05 was deemed significant.

## 3. Results

### 3.1. AgNPs Treatment Decreases the Tumor’s Global DNA Methylation

In the present work, we used tumor samples from the previous study in which 4T1 tumor-bearing mice were administered intragastrically (1 mg/kg b.w. daily, giving a total dose of 14 mg/kg b.w.) or intravenously (two administrations with 1 mg/kg b.w., giving a total dose of 2 mg/kg b.w.) with citrate- or PEG-coated AgNPs [22].

**Figure 1:**
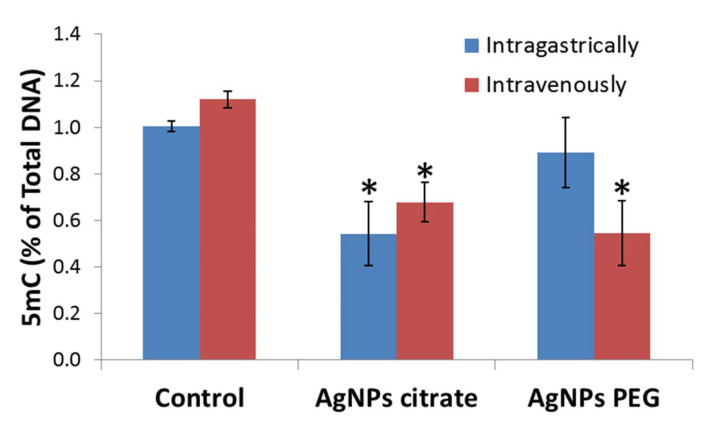
Level of 5-methylcytosine (5-mC) as a percent of total DNA in 4T1 tumors from control mice and mice that were administered citrate- or PEG-coated AgNPs via the intragastric or intravenous route. Means and standard deviations are shown. Asterisks represent statistically significant differences versus the control group. *n* = 3.

The analysis of global DNA methylation in the tumor tissue was performed. It revealed a significant, about two-fold, decrease in 5-methylcytosine (5-mC) level in tumors from mice treated with citrate-coated AgNPs regardless of the route of administration. For PEG-coated AgNPs, a significant decrease in DNA methylation was observed only after intravenous administration (Figure 1).

### 3.2. AgNPs Treatment Decreases Methylation but Not Acetylation of Histone H3 in Tumor Tissue

**Figure 2:**
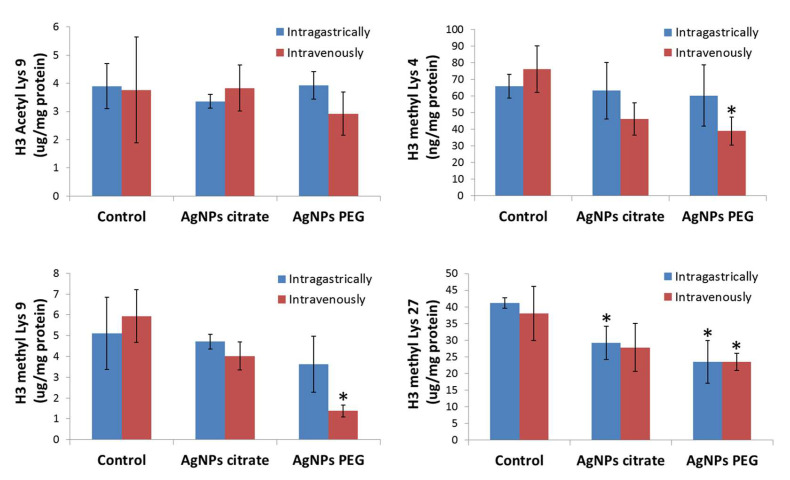
Level of histone H3 acetylation and methylation at Lys4, Lys9, and Lys27 in 4T1 tumors from control mice and mice that were administered citrate- or PEG-coated AgNPs via the intragastric or intravenous route. Means and standard deviations are shown. Asterisks represent statistically significant differences versus the control group. *n* = 3.

The next step of the analysis was the evaluation of acetylation and methylation levels of histone H3 in tumor samples. No changes in H3 Lys9 acetylation in tumors from mice treated with AgNPs relative to control animals were observed. However, treatment with PEG-coated AgNPs significantly decreased H3 Lys4 methylation in tumors. This effect was clearly dependent on the route of administration since it appeared only after intravenous AgNPs administration (Figure 2). A similar tendency was observed for citrate-coated AgNPs, but the effect was not statistically significant. 

Methylation of both Lys9 and Lys27 was significantly decreased in tumors from mice receiving PEG-coated AgNPs intravenously. However, after intragastric administration of this type of AgNPs, a significant methylation decrease was observed only at Lys27. A different pattern was observed for tumors explanted from the animals treated with citrate-coated AgNPs. In this case, a significant decrease in methylation level was observed only at Lys27 in tumors from mice treated intragastrically (Figure 2).

In general, treatment of 4T1 tumor-bearing mice with AgNPs decreased histone H3 methylation in tumor tissue and the effect was the most pronounced for PEG-coated AgNPs administered intravenously.

### 3.3. AgNPs Treatment Affects the Expression of Chromatin-Modifying Enzymes in Tumor Tissue

**Figure 3:**
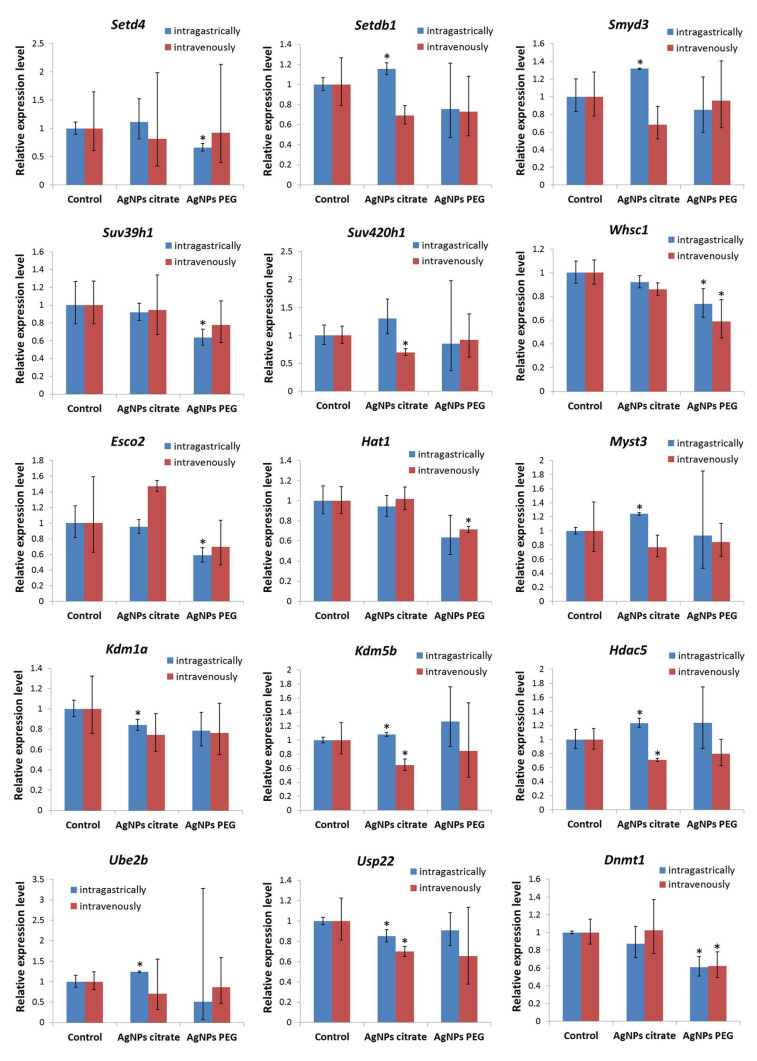
Relative mRNA expression level of enzymes responsible for chromatin modifications in tumors from mice treated intragastrically or intravenously with citrate- or PEG-coated AgNPs. Columns show mean fold changes. Whiskers represent minimum and maximum fold changes calculated from a standard deviation of ΔCt values. Asterisks represent statistically significant differences versus the control group. *n* = 3.

Gene expression analysis was performed to check if the observed decrease in DNA and histone H3 methylation was accompanied by changes in the expression of enzymes and factors responsible for chromatin modification. Results of the real-time PCR array analysis revealed 15 genes, whose expression in tumor tissue was affected by AgNPs treatment (Figure 3, Supplementary Table S1). Six of them encode methyltransferases (*Setd4*, *Setdb1*, *Smyd3*, *Suv39h1*, *Suv420h1*, and *Whsc1*), two encode demethylases (*Kdm1a* and *Kdm5b*), three encode acetyltransferases (*Esco2*, *Hat1*, and *Myst3*), one encodes histone deacetylase (*Hdac5*) [25,26], and one encodes DNA methylase (*Dnmt1*) [27]. Two genes encoding proteins involved in the ubiquitination process were also affected (*Ube2b* and *Usp22*) [28,29]. 

### 3.4. Effect of AgNPs Treatment on Expression of Oncogenes and Tumor Suppressor Genes in Tumor Tissue

**Figure 4:**
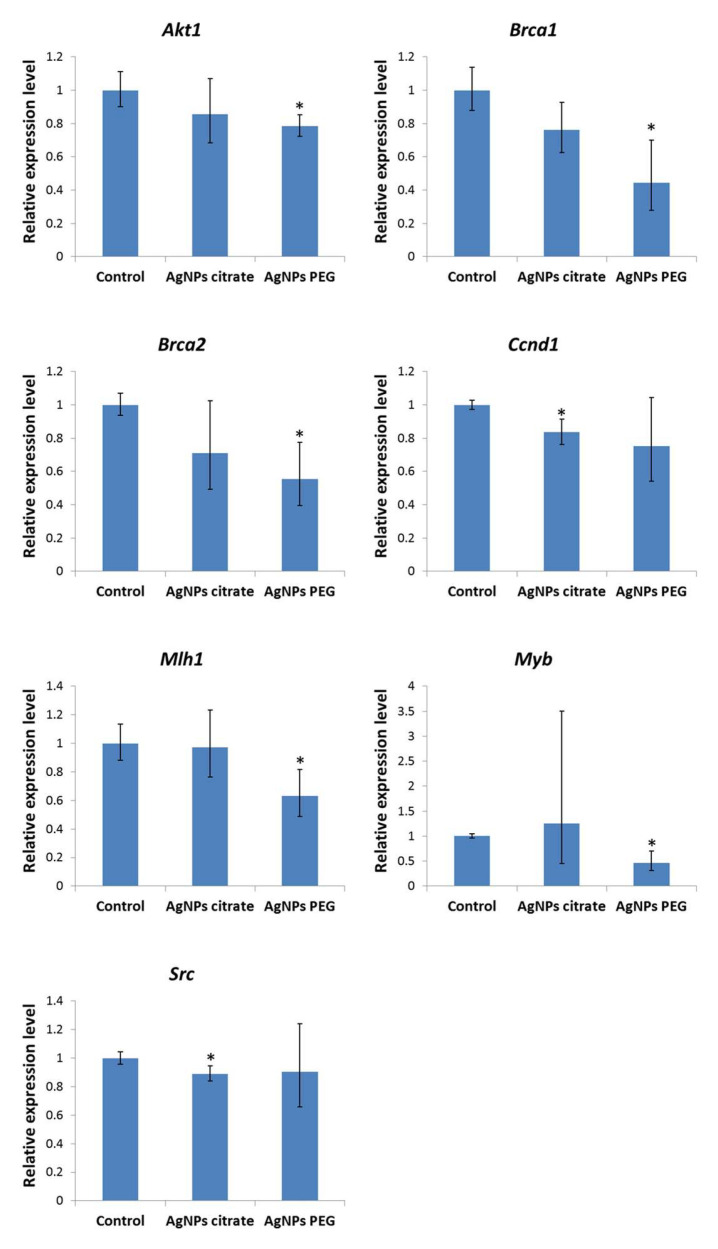
Relative mRNA expression level of oncogenes and tumor suppressor genes significantly deregulated in tumors from mice treated intragastrically with citrate- or PEG-coated AgNPs. Mean fold changes with minimum and maximum fold changes calculated from a standard deviation of ΔCt values are shown. Asterisks represent statistically significant differences versus the control group. *n* = 3.

Since methylation of DNA and histones is a powerful epigenetic mechanism regulating gene expression, we analyzed the possible correlation between changes in DNA and histone H3 methylation induced by AgNPs treatment and expression of oncogenes and tumor suppressor genes in the same samples. Real-time PCR array analysis revealed five genes down-regulated in tumors from mice treated with PEG-coated AgNPs (Akt1, Brca1, Brca2, Mlh1, and Myb) and two genes down-regulated in tumors from mice treated with citrate-coated AgNPs (Ccnd1 and Src) (Figure 4, Supplementary Table S2).

### 3.5. Expression of Metastasis-Related miRNAs in Tumor Tissue Was Not Affected by AgNPs Treatment

**Figure 5:**
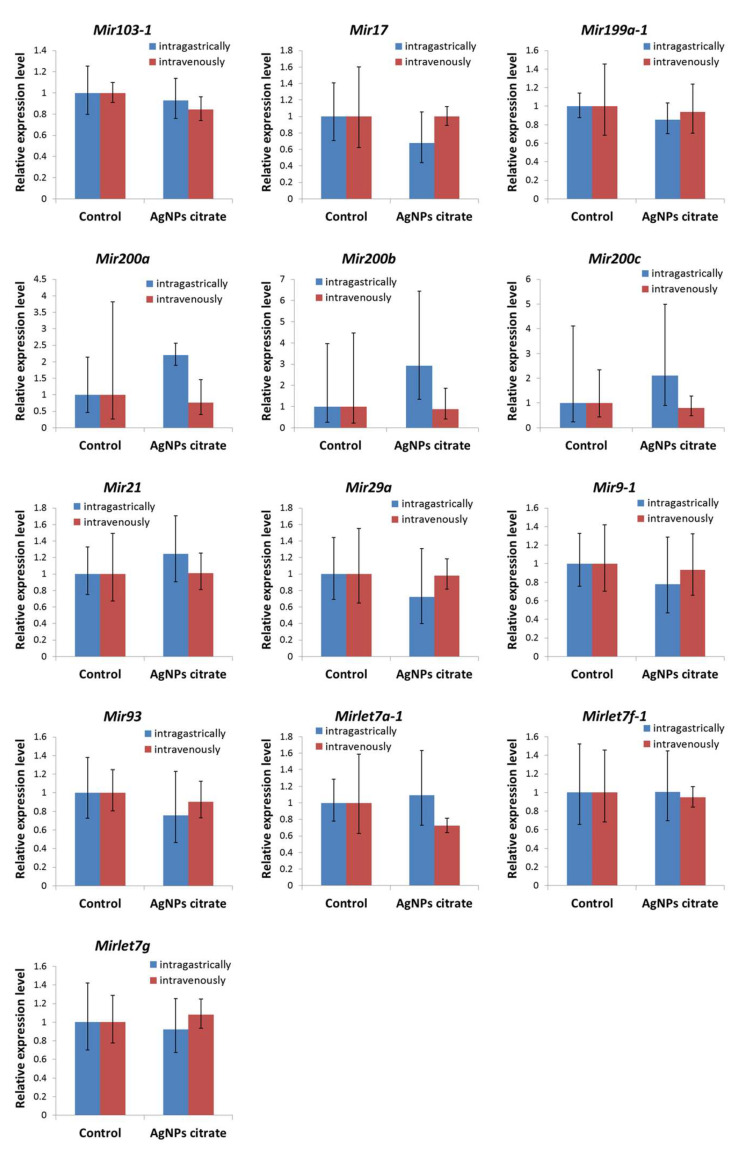
Relative expression level of miRNAs related to epithelial–mesenchymal transition and metastasis in tumors from mice treated intragastrically or intravenously with citrate-coated AgNPs. Mean fold changes with minimum and maximum fold changes calculated from a standard deviation of ΔCt values are shown. *n* = 3.

The expression of thirteen miRNAs related to EMT and metastasis was analyzed in the tumor tissue obtained from mice that received citrate-coated AgNPs and corresponding controls. No significant changes in miRNA expression were found (Figure 5).

## 4. Discussion

Epigenetic modifications are defined as stable and heritable alterations mainly controlled by three closely regulated and interconnected processes: DNA methylation, modification of histones, and expression of non-coding RNAs. Together, they alter chromatin structure and DNA accessibility, which results in the modulation of gene expression patterns [16]. It is important to keep in mind that the observed outcome of epigenetic changes is always a concerted action of epigenetic factors and many negative and positive feedback loops linking them.

Cytosine methylation is the most extensively studied epigenetic modification, predominantly associated with gene silencing. DNA methylation is a key factor in genomic imprinting since hypermethylation of one of the parental alleles leads to monoallelic expression [30]. DNA methylation is catalyzed by DNA methyltransferases, such as DNMT1 and DNMT3B. DNMT1 is primarily responsible for preserving methylation during DNA replication, whereas DNMT3B is involved in de novo DNA methylation [31]. Cancer cells exhibit a significant global reduction in DNA methylation (20–60% less overall 5-mC). At the same time, hypermethylation of the promoter regions of tumor suppressor genes is frequently observed [31]. In the present work, a decrease in global DNA methylation in tumors from mice treated with AgNPs was observed (Figure 1). This is in line with results published by other authors. For example, Maki et al. reported that citrate-coated AgNPs with a diameter of 10, 50, or 100 nm decreased the content of methylated DNA in A549 alveolar epithelial cells in vitro. This was accompanied by decreased level of Dnmt1 protein, while the level of Dnmt3b was significantly increased by 10 nm AgNPs treatment [32]. In line with these results, we observed a significant decrease in the expression of Dnmt1 in tumors from mice treated with AgNPs (Figure 3). However, this observation cannot serve as a full explanation of the detected decrease in DNA methylation, since it does not fully correlate with observed DNA methylation changes. While Dnmt1 expression decreased in mice treated with AgNPs both intragastrically and intravenously, DNA methylation decreased only after intravenous administration. Moreover, DNA methylation decreased in mice treated with citrate-coated AgNPs, but the Dnmt1 level remained unchanged. Apparently, the regulation of Dnmt1 activity is complex and is not limited to the transcription level. Maki et al. suggested that AgNPs induced DNA hypomethylation through proteasome-mediated degradation of Dnmt1 [32]. It seems that in our case more than one mechanism of Dnmt1 regulation was involved. In the present work, no significant changes in Dnmt3 expression in response to treatment with AgNPs occurred (Supplementary Table S1). Global DNA methylation and hydroxymethylation were also significantly reduced in the liver tissues of the AgNP-treated NAFLD (Non-alcoholic fatty liver disease) mice when compared with HFD (high-fat diet)-fed mice [33]. In another work, the effect of the 8 nm AgNPs was investigated in pregnant mice. The findings indicated that the AgNPs disturbed the progression of the first meiosis together with the expression of imprinted genes in placental tissue. In particular, the methylation of the Zac1 gene was significantly reduced in AgNP-treated placentas, while methylation of Igf2r was slightly increased in the AgNPs-treated group [9].

On the contrary, some experiments revealed increased global DNA methylation in A549, 293T, and HEK293T cells in vitro after treatment with sublethal concentrations of AgNPs [34]. In HEK293T cells, increased DNA methylation was accompanied by elevated levels of DNMT1 and DNMT3a proteins together with decreased expression of TET2 methylcytosine dioxygenase [20]. Accordingly, Blanco et al. reported increased DNA methylation in A549 cells treated with PVP-coated AgNPs; however, in this case, nanoparticles were used in a concentration that was toxic to the cells [35]. Elevated levels of 5-mc and DNMT enzymes were also observed in HT22 cells after treatment with 8 nm AgNPs at the EC50 [36].

Another very important epigenetic mechanism analyzed in the present study was the post-translational histone modification that is responsible for chromatin restructuration and in consequence transcription regulation. The analysis was focused on four important modifications of H3 histone, namely acetylation at Lys9 and methylation at Lys4, Lys9, and Lys27. Methylations at Lys9 and Lys27 are generally connected to transcription repression, whereas methylation at Lys4 and acetylation at Lys9 are enriched in the active gene regions [18]. Though we did not observe any changes in Lys9 acetylation, a tendency for a decrease in the methylation level of all three lysine residues under study was observed (Figure 2). The effect was the most pronounced for PEG-coated AgNPs administered intravenously. The literature on AgNPs-induced changes in histone modifications is scarce. In accordance with the results presented here, Qian et al. demonstrated that PVP-coated AgNPs reduced β-globin transcription by diminishing methylation of Lys4 and Lys79 in erythroid cells. The authors argued that the molecular mechanisms involved the decline in histone methyltransferase DOT-1L and MLL as well as a direct binding of AgNPs to histone that induced steric hindrance effects preventing methylation [37]. Another report described the deacetylation of histone H3 in A549 cells after treatment with PVP-coated AgNPs [35]. Wamucho et al. showed that exposure of soil nematode Caenorhabditis elegans to pristine AgNPs and its environmentally transformed product, sulfidized AgNPs, induced changes in histone H3 methylation. Methylation at Lys4 increased in response to pristine AgNPs exposure, whereas exposure to sulfidized AgNPs significantly decreased H3 methylation at Lys4 and Lys9 [38]. These three reports, together with the present study, suggest that the effect of AgNPs on histone modifications strongly depends on AgNPs formulation and the experimental model used. 

Histone modifications are regulated by various enzymes, including histone methyltransferases, demethylases, acetyltransferases, and deacetylases. To elucidate the mechanism responsible for observed changes in histone H3 methylation, the analysis of the expression of chromatin-modifying enzymes in tumor samples was performed (Figure 3, Supplementary Table S1). Observed changes in gene expression did not show a clear correlation with histone modification patterns. Though we observed changes in the expression of three histone acetyltransferases and one deacetylase, no changes in histone acetylation occurred. Similarly, even though changes in six genes encoding methyltransferases occurred, they did not correlate with observed changes in H3 methylation. One reason for this lack of clear correlation may be due to the fact that observed changes in gene expression are relatively low, not exceeding two folds. Therefore, their biological significance is questionable. On the other hand, such observations support the hypothesis of Qian et al. who postulated that decreased histone H3 methylation is (at least partially) the result of AgNPs binding to histone proteins hindering their modification by enzymes [37]. 

Epigenetic mechanisms, such as DNA methylation and histone modifications, regulate gene expression. In the previous study conducted on the same set of tumor samples, we did not find any significant changes in the expression of genes related to EMT and metastasis. Among genes related to inflammation, only Il12b was upregulated in tumor tissue collected from mice that received citrate-coated AgNPs intragastrically [22]. In the present study, dedicated PCR arrays were used to evaluate the expression level of a set of oncogenes and tumor suppressor genes. This part of the study was restricted to mice receiving AgNPs intragastrically, since only for this experimental group metastasis inhibition was observed. In this group of mice, decreased DNA methylation and decreased histone H3 methylation at Lys27 were observed (Figure 1 and Figure 2). Both modifications are linked with the repression of transcription; therefore, gene expression was expected to be up-regulated. Surprisingly, only seven genes were significantly affected and all of them were down-regulated (Figure 4). Moreover, a distinct set of genes were affected by citrate- and PEG-coated AgNPs, with the effect of PEG-coated nanoparticles, seeming to be more pronounced. Down-regulated genes belong to the class of oncogenes (Akt1, Ccnd1, Myb, and Src) and tumor suppressors (Brca1, Brca2, and Mlh1). These rather small changes in the expression of genes related to carcinogenesis are in agreement with the observation from our previous study that treatment with AgNPs has no effect on the kinetics of tumor growth [22].

The third epigenetic mechanism evaluated in the present study was miRNA expression. We have previously shown that nanomaterials, including AgNPs, can affect miRNA expression in vitro [21], which is in agreement with other studies [39,40,41]. Here, the analysis is focused on miRNAs that are known to be related to EMT and metastasis in the hope of finding possible epigenetic mechanisms responsible for observed metastasis inhibition by AgNPs. However, no changes in miRNAs expression have been found in the tumor tissue collected from mice that received AgNPs (Figure 5), which supports the hypothesis formulated in our previous paper stating, that AgNPs-mediated metastasis inhibition was not the result of nanoparticles’ direct impact on cancer cells but rather was a consequence of AgNPs-induced modulation of the immune system activity [22].

In conclusion, we have shown that AgNPs affect epigenetic mechanisms of cellular regulation such as DNA and histone methylation, as well as gene expression in tumor tissue in vivo. However, the significance of the observed changes and the mechanisms responsible for their occurrence are unclear, and more research in this area is warranted. Nevertheless, the findings of the present study are important for the biomedical field, as they point to the epigenetic effects as an important level of interaction between nanomaterials and biological systems. Consequently, epigenetic effects induced by nanomaterials should always be taken into consideration not only during nanotoxicological studies but also during the development and evaluation of novel, nanoparticle-based anticancer therapies.

## Data Availability

The raw data are available upon request from the corresponding author.

## Supplementary Materials

The following supporting information can be downloaded at: https://www.mdpi.com/article/10.3390/ma16114163/s1, Table S1: Expression of chromatin modification enzymes at the mRNA level in tumors from mice treated intragastrically or intravenously with citrate- or PEG-coated AgNPs. Rq- mean expression level relative to control. Statistically significant changes relative to control are highlighted; Table S2: Expression of oncogenes and tumor suppressor genes at the mRNA level in tumors from mice treated intragastrically with citrate- or PEG-coated AgNPs. Rq- mean expression level relative to control. Statistically significant changes relative to control are highlighted.
