# Supplementary material for: Silver Nanoparticles Induced Changes in DNA Methylation and Histone H3 Methylation in a Mouse Model of Breast Cancer
Source: Materials (Basel). 2023 Jun 2;16(11):4163. doi: 10.3390/ma16114163 (PMC10254218; doi:10.3390/ma16114163)
Supplement: Supplementary file 1 [file materials-16-04163-s001.zip › materials-2371863-supplementary.pdf]

Supplementary Table S1. Expression of chromatin modification enzymes at the mRNA level in tumors from mice treated intragastrically or intravenously with citrate- or PEG-coated AgNPs. Rq- mean expression level relative to control. Statistically significant changes relative to control are highlighted.

Target Name	Intragastric administration				Intravenous administration			
	AgNPs citrate		AgNPs PEG		AgNPs citrate		AgNPs PEG	
	Rq	P-Value (t-test)	Rq	P-Value (t-test)	Rq	P-Value (t-test)	Rq	P-Value (t-test)
Ash1l	1.053	0.222	0.845	0.476	0.702	0.107	0.747	0.348
Atf2	1.115	0.374	0.940	0.768	0.776	0.202	0.892	0.632
Aurka	1.008	0.969	1.211	0.690	1.322	0.490	1.212	0.627
Aurkb	0.956	0.815	0.933	0.876	1.285	0.441	1.024	0.951
Carm1	1.015	0.838	0.955	0.688	0.812	0.154	0.891	0.676
Cdyl	0.904	0.251	0.933	0.522	0.972	0.859	0.957	0.823
Ciita	1.989	0.116	1.326	0.660	1.366	0.711	2.469	0.347
Csrp2bp	1.134	0.077	0.881	0.566	0.880	0.624	1.183	0.562
Dnmt1	0.875	0.258	0.611	0.005	1.096	0.902	0.665	0.026
Dnmt3a	1.170	0.617	2.293	0.132	0.870	0.734	1.620	0.302
Dnmt3b	0.915	0.550	0.608	0.073	0.736	0.549	1.119	0.815
Dot1l	0.945	0.695	0.863	0.560	0.794	0.165	0.731	0.164
Dzip3	1.140	0.418	0.877	0.630	0.826	0.319	0.698	0.208
Ehmt1	0.981	0.886	0.701	0.144	0.618	0.271	0.730	0.437
Ehmt2	0.956	0.613	0.871	0.437	0.681	0.339	0.823	0.578
Esco1	1.244	0.228	2.102	0.156	1.112	0.156	1.563	0.077
Esco2	0.954	0.701	0.588	0.015	1.473	0.181	0.697	0.312
Hat1	0.943	0.553	0.633	0.054	1.019	0.845	0.714	0.009
Hdac1	0.987	0.859	0.406	0.439	0.804	0.251	0.843	0.260
Hdac10	1.528	0.382	2.969	0.234	1.150	0.688	2.406	0.148
Hdac11	1.329	0.375	1.221	0.412	0.654	0.477	1.188	0.778
Hdac2	0.923	0.204	0.864	0.380	0.746	0.255	0.689	0.182
Hdac3	0.950	0.540	0.816	0.089	0.795	0.093	0.855	0.486
Hdac4	1.105	0.396	0.961	0.815	0.710	0.132	0.987	0.951
Hdac5	1.234	0.046	1.235	0.330	0.710	0.011	0.796	0.180
Hdac6	0.918	0.204	0.795	0.273	0.707	0.107	0.777	0.286
Hdac7	1.120	0.581	1.062	0.828	0.890	0.609	1.271	0.287
Hdac8	1.157	0.285	0.925	0.778	0.821	0.457	1.255	0.375
Hdac9	1.303	0.548	0.909	0.772	1.206	0.762	2.563	0.221
Kat2a	1.048	0.688	0.944	0.790	0.722	0.236	0.798	0.479
Kat2b	1.302	0.331	2.717	0.134	0.861	0.427	1.472	0.203
Kat5	1.061	0.518	1.537	0.280	0.813	0.330	1.074	0.741
Kdm1a	0.842	0.031	0.783	0.100	0.745	0.196	0.762	0.281
Kdm4a	0.901	0.421	0.746	0.140	0.633	0.353	0.849	0.692
Kdm4c	1.095	0.165	0.985	0.921	0.758	0.226	0.813	0.340

Kdm5b	1.077	0.042	1.265	0.237	0.644	0.027	0.849	0.636
Kdm5c	1.239	0.099	0.964	0.883	0.870	0.479	1.190	0.359
Kdm6b	1.118	0.451	0.956	0.734	0.547	0.126	0.726	0.246
Mll3	1.098	0.530	0.946	0.807	0.710	0.460	1.133	0.784
Mll5	1.109	0.547	1.101	0.767	0.759	0.140	0.839	0.508
Mysm1	1.090	0.377	0.825	0.404	0.704	0.053	0.795	0.239
Myst2	0.885	0.100	0.737	0.104	0.751	0.189	0.688	0.141
Myst3	1.241	0.001	0.935	0.858	0.770	0.265	0.842	0.484
Myst4	0.985	0.477	0.944	0.781	0.621	0.231	0.797	0.528
Ncoa1	1.090	0.651	1.038	0.916	0.686	0.180	1.185	0.594
Ncoa3	1.172	0.290	1.139	0.682	0.946	0.889	1.441	0.367
Ncoa6	1.115	0.161	0.974	0.871	0.758	0.075	0.730	0.074
Nek6	1.173	0.200	0.992	0.982	0.700	0.203	0.826	0.666
Nsd1	1.007	0.969	0.770	0.241	0.835	0.684	1.108	0.824
Pak1	1.081	0.592	0.854	0.486	1.032	0.915	0.770	0.268
Prmt1	0.860	0.146	1.189	0.517	0.906	0.258	0.907	0.440
Prmt2	1.112	0.355	1.001	0.992	0.720	0.165	0.732	0.436
Prmt3	0.938	0.654	0.711	0.096	0.830	0.508	0.721	0.117
Prmt5	0.937	0.249	0.716	0.099	0.897	0.251	0.728	0.159
Prmt6	1.147	0.541	2.382	0.162	0.830	0.310	1.258	0.324
Prmt7	1.006	0.958	0.847	0.265	0.827	0.398	0.892	0.680
Prmt8	1.088	0.817	2.057	0.142	0.299	0.148	0.824	0.845
Rnf2	0.986	0.903	0.973	0.834	0.777	0.123	0.782	0.264
Rnf20	1.098	0.508	0.878	0.517	0.837	0.315	1.005	0.973
Rps6ka3	1.003	0.961	0.889	0.577	0.904	0.307	1.043	0.756
Rps6ka5	1.186	0.131	0.932	0.698	0.731	0.115	0.837	0.377
Setd1a	1.053	0.816	0.557	0.454	0.790	0.292	0.846	0.466
Setd1b	0.995	0.980	0.584	0.484	0.584	0.175	0.782	0.559
Setd2	1.061	0.299	0.814	0.512	0.712	0.141	0.791	0.325
Setd4	1.116	0.552	0.664	0.006	0.816	0.715	0.923	0.879
Setd5	0.956	0.651	0.835	0.386	0.730	0.200	0.836	0.460
Setd6	1.053	0.808	1.769	0.221	0.830	0.359	1.240	0.317
Setd7	0.999	0.998	0.709	0.185	0.632	0.404	0.637	0.410
Setd8	0.894	0.374	0.810	0.193	0.967	0.868	0.797	0.270
Setdb1	1.157	0.026	0.758	0.318	0.691	0.055	0.728	0.251
Setdb2	1.134	0.300	0.786	0.291	1.081	0.497	1.211	0.609
Smyd1	1.013	0.985	0.580	0.636	3.149	0.288	2.147	0.401
Smyd3	1.317	0.042	0.852	0.482	0.685	0.111	0.955	0.855
Suv39h1	0.920	0.562	0.634	0.031	0.946	0.806	0.778	0.265
Suv420h1	1.307	0.148	0.855	0.739	0.698	0.016	0.921	0.730
Tbp	1.011	0.915	0.838	0.327	0.816	0.289	0.864	0.479
Ube2a	1.043	0.516	0.713	0.373	0.826	0.409	0.794	0.271
Ube2b	1.244	0.043	0.504	0.514	0.708	0.452	0.867	0.687

Usp16	1.181	0.184	0.594	0.453	0.750	0.179	0.931	0.740
Usp21	1.245	0.320	2.462	0.121	0.844	0.669	1.679	0.167
Usp22	0.852	0.017	0.908	0.346	0.699	0.031	0.654	0.229
Whsc1	0.922	0.221	0.736	0.034	0.859	0.065	0.589	0.023

Supplementary Table S2. Expression of oncogenes and tumor suppressor genes at the mRNA level in tumors from mice treated intragastrically with citrate- or PEG-coated AgNPs. Rq- mean expression level relative to control. Statistically significant changes relative to control are highlighted.

Target Name	AgNPs citrate		AgNPs PEG	
	Rq	P-Value (t-test)	Rq	P-Value (t-test)
Abl1	0.920	0.169	0.852	0.097
Akt1	0.856	0.288	0.785	0.024
Apc	1.192	0.160	0.782	0.471
Atm	1.289	0.198	0.775	0.244
Bax	0.977	0.818	1.023	0.920
Bcl2	1.282	0.579	2.508	0.074
Bcl2l1	0.922	0.707	1.013	0.972
Bcr	1.061	0.651	0.940	0.681
Brca1	0.762	0.085	0.443	0.029
Brca2	0.711	0.147	0.555	0.028
Casp8	1.068	0.321	0.985	0.970
Ccnd1	0.836	0.021	0.752	0.167
Cdh1	0.938	0.931	0.811	0.664
Cdk4	0.902	0.244	1.107	0.647
Cdkn1a	0.993	0.982	1.196	0.699
Cdkn3	0.942	0.748	0.565	0.130
Ctnnb1	0.896	0.387	1.071	0.800
E2f1	0.973	0.738	0.705	0.067
Elk1	0.808	0.533	0.940	0.839
Erbb2	1.215	0.506	1.288	0.351
Esr1	0.956	0.835	0.916	0.709
Ets1	1.134	0.331	0.759	0.292
Fos	0.415	0.106	1.191	0.758
Foxd3	1.839	0.548	5.677	0.167
Hgf	1.530	0.385	1.387	0.570
Hic1	1.378	0.672	3.369	0.168
Hras1	2.375	0.447	7.008	0.175
Igf2r	1.112	0.454	1.079	0.508
Jak2	1.317	0.462	1.040	0.825
Jun	0.902	0.711	1.348	0.377
Junb	1.061	0.865	1.975	0.297
Jund	1.650	0.538	3.647	0.180
Kit	1.111	0.658	1.377	0.135
Kitl	1.353	0.308	1.219	0.549
Kras	0.914	0.126	0.901	0.230
Mcl1	1.066	0.177	1.056	0.497
Mdm2	0.886	0.469	0.777	0.061

Men1	0.900	0.675	0.831	0.316
Met	0.879	0.096	0.650	0.053
Mgmt	1.114	0.426	1.343	0.060
Mlh1	0.972	0.845	0.630	0.035
Mos	2.791	0.419	7.898	0.178
Myb	1.254	0.645	0.467	0.023
Myc	1.154	0.294	1.066	0.826
Mycn	2.309	0.152	1.832	0.257
Nf1	1.087	0.096	0.891	0.431
Nf2	0.880	0.201	0.793	0.106
Nfkb1	0.945	0.691	0.721	0.378
Nfkbia	1.029	0.912	0.954	0.867
Nras	0.964	0.841	0.821	0.183
Pik3c2a	1.084	0.402	1.038	0.810
Pik3ca	1.115	0.476	1.039	0.865
Pml	1.187	0.107	0.895	0.519
Prkca	1.075	0.514	1.033	0.856
Raf1	1.031	0.476	0.893	0.234
Rara	0.621	0.258	1.244	0.641
Rassf1	0.990	0.950	1.198	0.281
Rb1	1.038	0.796	0.986	0.918
Rel	1.254	0.161	0.953	0.600
Ret	0.837	0.508	0.624	0.361
Runx1	1.017	0.792	1.186	0.059
Runx3	0.770	0.268	0.622	0.093
S100a4	0.922	0.248	0.500	0.111
Serpinb5	0.391	0.451	0.702	0.768
Sh3pxd2a	0.893	0.388	0.920	0.722
Smad4	1.067	0.539	0.882	0.509
Src	0.890	0.037	0.903	0.567
Stat3	1.122	0.449	1.227	0.208
Stk11	1.015	0.720	1.045	0.870
Tbp	0.983	0.900	0.850	0.547
Tgfb1	1.036	0.507	0.949	0.749
Tnf	2.101	0.435	5.841	0.180
Trp53	0.698	0.212	0.731	0.288
Tsc1	1.211	0.430	1.038	0.793
Vhl	0.916	0.499	0.703	0.060
Wt1	0.753	0.530	0.808	0.720
Wwox	0.742	0.069	0.752	0.297
Xrcc1	0.792	0.336	1.172	0.519
Zhx2	1.220	0.292	0.989	0.974
